# P-650. *In vitro* Activity of Cefiderocol and Comparator Agents Against Global Enterobacterales Isolates From Hospitalized Patients with Pneumonia Collected as Part of the SENTRY Surveillance Program (2020-2022)

**DOI:** 10.1093/ofid/ofae631.847

**Published:** 2025-01-29

**Authors:** Jason J Bryowsky, Boudewijn L DeJonge, Sean T Nguyen, Joshua Maher, Rodrigo E Mendes, Christopher M Longshaw, Miki Takemura, Yoshinori Yamano

**Affiliations:** Shionogi Inc., Florham Park, New Jersey; Shionogi Inc., Florham Park, New Jersey; Shionogi Inc., Florham Park, New Jersey; JMI Laboratories, North Liberty, Iowa; JMI Laboratories, North Liberty, Iowa; Shionogi B.V., London, England, United Kingdom; Shionogi & Co., Ltd, Toyonaka, Osaka, Japan; Shionogi & Co., Ltd., Toyonaka, Osaka, Japan

## Abstract

**Background:**

The *in vitro* activity of cefiderocol (CFDC) and comparator agents was evaluated against Enterobacterales isolates collected from patients with pneumonia during 2020-2022 as part of the SENTRY Antimicrobial Surveillance Program.

**Figure:**
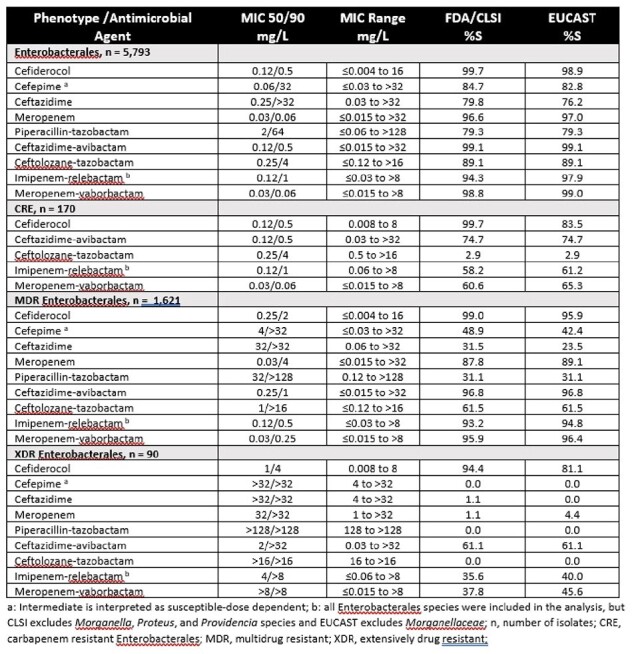

**Methods:**

5,793 Enterobacterales (40.5% *Klebsiella* spp., 19.6% *E. coli*, 13.6% *Serratia* spp., 11.6% *Enterobacter* spp., 5.1% *Proteus* spp., 5% *Citrobacter* spp., 4.6% other) were collected from pneumonia patients and tested for susceptibility (%S) using CLSI broth microdilution with cation-adjusted Mueller-Hinton broth (CAMHB) or iron-depleted CAMHB for CFDC. Comparator agents included β-lactam/β-lactamase inhibitor (BL/BLI) combinations ceftazidime-avibactam (CZA), ceftolozane-tazobactam (C/T), imipenem-relebactam (I-R) meropenem-vaborbactam (M-V), piperacillin-tazobactam as well as meropenem (MEM), cefepime and ceftazidime . Susceptibility was interpreted according to 2024 CLSI|FDA, and EUCAST breakpoints. Carbapenem resistant Enterobacterales (CRE) was defined as resistant to imipenem and MEM. Multidrug-resistant (MDR) Enterobacterales was defined as nonsusceptible to at least 1 drug from ≥3 classes and extensively drug resistant (XDR) as susceptible to ≤2 classes per 2024 CLSI criteria.

**Results:**

47% and 53% of isolates were from North America and Europe, respectively. CFDC, MEM, CZA, I-R and M-V all showed high susceptibility against all Enterobacterales isolates ( >94/ >97 %S using CLSI|FDA/EUCAST breakpoints). Against CRE, CFDC (99.7/83.5 %S CLSI|FDA/EUCAST) was the most active agent while CZA was the second most active (74.7%S CLSI|FDA and EUCAST). CFDC, CZA, I-R, M-V were most active against MDR isolates ( >93/ >94 %S CLSI|FDA/EUCAST) however, CFDC (94.4/81.1 %S CLSI|FDA/EUCAST) was the only agent with appreciable activity against XDR isolates.

**Conclusion:**

CFDC demonstrated high *in vitro* activity against Enterobacterales isolated from hospitalized patients with pneumonia, including MDR and XDR isolates. Susceptibility for comparator agents, comprising BL/BLI combinations, was generally lower against isolates with CRE and XDR phenotypes. CFDC represents an important treatment option for pneumonia infections caused by Enterobacterales with presumed or defined CRE, MDR, or XDR phenotypes.

**Disclosures:**

**Jason J. Bryowsky, PharmD, MS**, Shionogi: Employee **Boudewijn L. DeJonge, PhD**, Shionogi Inc.: Employee **Sean T. Nguyen, PharmD**, Shionogi Inc.: Employee **Rodrigo E. Mendes, PhD**, GSK: Grant/Research Support **Christopher M. Longshaw, PhD**, Shionogi BV: Employee **Miki Takemura, n/a**, Shionogi & Co., Ltd.: Employee **Yoshinori Yamano, PhD**, Shionogi & Co., Ltd.: Employee

